# Taking up the reins of power: metabolic functions of p53

**DOI:** 10.1093/jmcb/mjz065

**Published:** 2019-08-15

**Authors:** Timothy Humpton, Karen H Vousden

**Affiliations:** 1 The CRUK Beatson Institute, Glasgow G61 1BD, UK; 2 The Francis Crick Institute, London NW1 1AT, UK

The clear importance of p53 as a tumour suppressor protein has propelled decades of intense research focused on understanding the functions of p53 and attempting to harness this knowledge for patient benefit. p53 plays a pivotal role in the ability of cells to sense and respond to stress—functions that contribute not only to limitation of cancer development, but also to modulating numerous other aspects of health and disease. Although the canonical activities of p53 relate to the elimination of damaged cells through cell death or senescence, more recent work has highlighted a role for p53 as a guardian of cell survival and facilitator of adaptation during metabolic stress. These emerging metabolic features of p53 activity are proving to be crucial for many of its essential functions.

Metabolic alterations have been associated with cancer development since Warburg noted high glucose uptake and lactate production in many cancer types. Subsequent studies have shown that tumour cells change metabolism in order to balance the needs of anabolism and energy generation—to allow for proliferation and dissemination—with the requirement to survive in abnormal, hostile, and nutrient-variable conditions (Pavlova and Thompson, 2016). Unsurprisingly, perhaps, given the breadth of diversity across human tissue niches, the metabolic requirement of tumours depends not only on the underlying genetic alterations driving the oncogenic process, but also on the tissue of tumour origin, the stage of cancer progression, the host environment, and the host immune state (Mayers and Vander Heiden, 2017). Adaptation to such heterogeneity is provided by the malleability of tumour metabolic responses. This exceptional plasticity creates significant challenges as we attempt to therapeutically intervene in these pathways.

As our understanding of metabolic alterations in cancers has expanded, so have the roles of p53 in mediating these responses (Labuschagne et al., 2018). Proliferating tumour cells depend on anabolic pathways to produce biomass. Consequently, cancer cells often activate glycolysis to enhance nucleotide production and augment fatty acid synthesis. Befitting a tumour suppressor, several of the metabolic functions of p53 oppose the metabolic changes commonly acquired during tumorigenesis. For example, p53 can limit or inhibit glycolysis—so preventing the glycolytic phenotype characteristic of many cancer cells (Zawacka-Pankau et al., 2011). Flux through the pentose phosphate pathway, which produces ribose that is essential for nucleotide synthesis, can also be limited by p53 (Jiang et al., 2011). p53 also regulates various aspects of lipid metabolism, such as by activating the expression of Abca1, so preventing the maturation of the transcription factor SREBP2—an important activator of mevalonate pathway genes (Moon et al., 2019). As a result, loss of p53 leads to increased mevalonate pathway activity that contributes to liver cancer development. p53 also plays a role in the regulation of the urea cycle, leading to an accumulation of ammonia and the suppression of translation of ornithine decarboxylase, a rate-limiting enzyme of polyamine synthesis (Li et al., 2019). A consequence of this activity of p53 is to limit tumour proliferation and growth. *De novo* serine synthesis—a pathway that becomes important in many cancers—is directly impaired by p53-mediated inhibition of PHGDH, the rate-limiting enzyme in this pathway (Ou et al., 2015). Furthermore, p53 can play a role in eliminating cells undergoing metabolic stress by enhancing the sensing of extracellular adenosine, which accumulates under these conditions (Long et al., 2013). In these situations, metabolic functions of p53 easily map onto the p53-tumour suppressor framework.

Even though tumour cells ramp up various metabolic pathways to support growth and proliferation, an expanding tumour mass can still become starved of nutrients, a situation that may not be entirely resolved by the development of leaky and ill-formed tumour vasculature. Cancer cells therefore develop numerous mechanisms to allow them to survive nutrient and oxygen starvation and surprisingly, under some conditions, p53 can contribute to these supportive responses (Humpton and Vousden, 2016). Induction of pathways such as autophagy, macropinocytosis, and endocytosis allow scavenging of nutrients from the microenvironment or through a limited degree of self-cannibalization. p53 has been shown to activate autophagy—a process that in the short term, at least, can help to recycle nutrients and keep cells alive (Rabinowitz and White, 2010)—and clearly supports cancer development (Guo and White, 2016). Intriguingly, the contribution of autophagy to tumour development in the pancreas depends on the retention of wild-type p53 (Rosenfeldt et al., 2013).

Nutrient depletion can cause an energetic crisis and so engage AMPK, a critical energy-sensing protein (Hardie, 2014). In response to diminished ATP, AMPK promotes catabolic pathways that produce ATP, while limiting anabolic energy-utilizing pathways, so supporting cell survival. In many ways, there is a close thematic relationship between p53 and AMPK, and this is enhanced by the fact that each can induce and respond to the other (Feng et al., 2007). Like AMPK, p53 can also limit energy-consuming pathways, like fatty acid synthesis, while promoting energy-producing pathways such as fatty acid oxidation or mitochondrial metabolism (Humpton and Vousden, 2016). Furthermore, p53 can help to conserve cell viability under nutrient-limiting conditions through the induction of cell cycle arrest, a response that limits the anabolic demands that accompany proliferation. Key to this response is the activation of the cyclin-dependent kinase inhibitor p21, expression of which can help to retard or promote tumorigenesis (Warfel and El-Deiry, 2013). Importantly, although p21 expression can contribute to the induction of an irreversible proliferative arrest, transient induction of p21 is generally reversible, allowing cells to re-enter the cell cycle once stress or damage has been resolved. This ability to turn off the p53 response would seem to be critical to allow for recovery at the end of a period of metabolic stress. Further mechanisms underlying p53 pro-survival functions in nutrient-depleted conditions are still being elucidated, and some may vary according to which nutrient is limiting. For example, many tumours develop a microenvironment that is selectively depleted of glutamine (Kamphorst et al., 2015). The cell’s ability to survive glutamine starvation is bolstered by the expression of p53-dependent genes, including the glutamate/aspartate transporter Slc1a3, which can support the malate-aspartate shuttle (Tajan et al., 2018), and Slc7a3, which enhances arginine import (Lowman et al., 2019). Manipulating glucose levels instead of glutamine engages different p53 pathways to promote survival (Jones et al., 2005). For example, p53-dependent activation of the long noncoding RNA TRINGS (Khan et al., 2017) and p53 induction of Acad11 (Jiang et al., 2015a)—a protein involved in fatty acid oxidation—can both support survival under conditions of glucose starvation.

Another challenge that is commonly encountered by tumour cells is excessive oxidative stress, resulting from the activation of oncogenes, perturbed metabolism, and loss of normal environmental support (Gorrini et al., 2013). In response, tumour cells activate anti-oxidant defence mechanisms to support their survival. Several p53 functions can promote ROS and so tip cancer cells into death. These include the transcriptional activation of pro-oxidant genes (Liu and Xu, 2011) and inhibition of the pathways that produce NADPH (Jiang et al., 2011; Jiang et al., 2013), a cofactor essential for the recycling of the antioxidant glutathione. p53 also represses the expression of Slc7a11, a component of the cystine/glutamate antiporter xCT (Jiang et al., 2015b). Reduction in cystine uptake through this mechanism limits the antioxidant capacity of cells and can drive the ROS and iron-dependent cell death program known as ferroptosis. Indeed, several functions of p53 have recently been shown to promote ferroptosis (Gnanapradeepan et al., 2018). But the regulation of oxidative stress is an area in which the dichotomous activities of p53 are clearly evident. In addition to promoting ROS, p53 can function to limit ROS to help promote cell survival. A number of antioxidant p53 target genes have been identified (Liu and Xu, 2011) including several that promote NADPH production through increased activity of the oxidative arm of the pentose phosphate pathway (Bensaad et al., 2006; Duan et al., 2018) or promote the production of the antioxidant GSH (Hu et al., 2010; Suzuki et al., 2010). Moreover, albeit less directly, p53 can also play a role in maintaining mitochondrial integrity and function (Itahana and Itahana, 2018), with loss of p53 resulting in less efficient oxidative phosphorylation and increased ROS. Induction of p53 has also been shown to protect cells from ferroptosis (Xie et al., 2017; Tarangelo et al., 2018). It is clear that the redox control functions of p53 are complex and often contradictory.

We are therefore left with evidence that p53 can function in seemingly opposing ways in response to nutrient stress. On the one hand, p53 can increase ROS and promote cell elimination and death. On the other, p53 can foster adaptation to nutrient deprivation, protect cells from ROS, and support survival. How to integrate these multiple functions of p53 is a topic of great interest. A simple model would suggest that p53 helps to support cell adaptation and survival in response to transient and/or reversible stress such as limited periods of nutrient starvation, while driving the elimination of cells exposed to irreparable stress-induced damage (Figure 1). Whether this model is correct is not entirely clear and many questions remain unanswered. For one, how is the switch from cell survival to cell death mediated? It is possible that context-specific collateral signals within a stress state act to modulate the p53 response itself, by directing different transcriptional programmes that would induce cell survival over cell death genes. Alternatively, or additionally, p53-independent signals may cooperate with p53 to tip the balance appropriately. Elegant studies from many years ago showed that simply dialling up the amount of p53 could switch cells from cell cycle arrest (a potentially reversible state that contributes to many of the survival scenarios) to cell death (Chen et al., 1996). These studies suggest the existence of a ‘goldilocks zone’ where just the right amount of p53 signalling supports survival, while too much induces death. More research will be required to reveal the molecular underpinnings of such a balancing act. As interesting as uncovering how p53 toggles between functions is to determine what the evolutionary selection for these different functions of p53 might be and what impact tumour-associated p53 mutations have on these activities.

**Figure 1:**
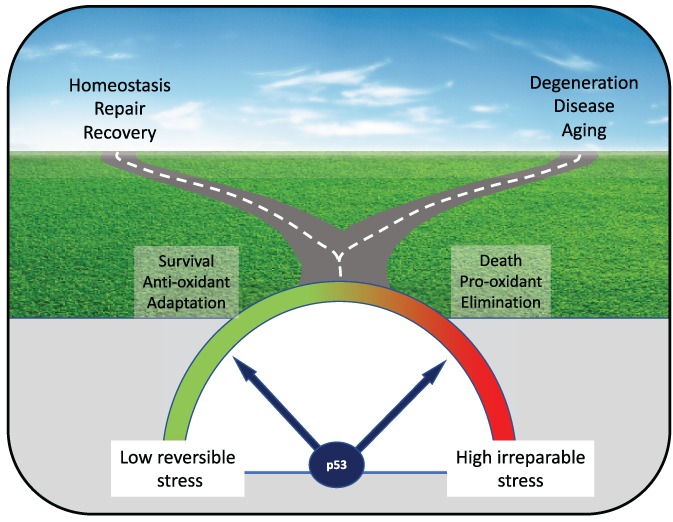
p53 can induce divergent paths to support survival or promote death. Depending on the nature, intensity, and duration of applied stress, p53 engages programs to support cell survival and adaptation or cause death and promote cell elimination. The model suggests that p53 can aid in recovery and repair under conditions of mild and reversible stress, but acts to eliminate cells exposed to persistent stress or irreversible damage. While both responses have the potential to protect from cancer development, inappropriate activation of the death response can promote degenerative diseases or early aging, while inappropriate maintenance of the survival response could help to support malignant development.

Tumour-derived mutations in p53 frequently give rise to a single-point mutation leading to the expression of a mutant protein. In general, the commonly occurring missense mutations in p53 result in a significant loss of wild-type p53 transcriptional activity, either by altering a DNA-contacting residue or by changing the structure of the protein entirely (Freed-Pastor and Prives, 2012). These alterations consistently ablate the ability of these p53 mutants to promote cell death or cell elimination—an observation that clearly implicates these functions in effective tumour suppression. These mutant p53s can also induce metabolic responses that are opposite to those triggered by wild-type p53. For example, mutant p53 can activate glycolysis (Zhang et al., 2013) and suppress mitochondrial metabolism (Eriksson et al., 2017)—although these activities can vary depending on mutation and tissue type. Mutant p53 can also promote the mevalonate pathway, so allowing cells to survive under conditions of matrix detachment (Freed-Pastor et al., 2012). Intriguingly, some tumour-associated p53 mutants show selective retention of wild-type functions that contribute to survival under nutrient and oxidative stress (Tran et al., 2017; Humpton et al., 2018). Maintenance of both p21 and MDM2 expression by the hotspot p53 mutant R248, for example, results in an ability to survive glutamine and serine starvation, although this p53 mutant is not able to induce cell death or senescence. This leads to the interesting possibility that expression of point mutations that selectively retain wild-type p53 survival functions would be selected more strongly during tumour evolution than mutations that abolish p53 entirely. In support of this suggestion, patients carrying tumours with R248 p53 mutants show a particularly poor survival rate (Xu et al., 2014; Humpton et al., 2018), even compared to patients with tumours harbouring other common p53 mutations.

Finally, and moving away from cancer, we should consider whether the metabolic functions of p53 have been selected for purposes other than tumour surveillance. p53 is now being implicated in numerous aspects of health and disease—in several cases reflecting the negative consequences of cell death following p53 activation. In ischemia, for example, inhibition of p53 is likely to have therapeutic benefit (Gudkov and Komarova, 2010). The role of p53 in the development of obesity (a consequence of nutrient excess) and metabolic syndrome is not yet clear, although some studies have shown that the retention of p53 activity can support weight gain (Derdak et al., 2013; Porteiro et al., 2013). While this is a somewhat detrimental response in the 21st century, it is likely to have provided a strong selective advantage in the past. Other clear benefits to maintaining wild-type p53 activity beyond tumour suppression are also beginning to emerge. In normal reproduction, for example, p53 modulates the level and function of LIF, a cytokine critical for embryo implantation and subsequent normal progression through pregnancy (Hu et al., 2007). In terms of metabolism, the ability of p53 to maintain mitochondrial health is associated with increased stamina during exercise (Park et al., 2009), while p53-mediated regulation of lipid metabolism may help to promote liver homeostasis and protect from liver steatosis (Liu et al., 2014; Prokesch et al., 2017). We note, however, that the role of p53 in the development of liver disease and obesity is highly complex and discussed in more detail elsewhere (Labuschagne et al., 2018). Chronic p53 activation can also lead to early aging, although the enhanced ability to appropriately activate p53 may improve lifespan (Wu and Prives, 2018). While p53 activity is dramatically altered in many cancers following mutation, p53 function is also more subtly modulated via the retention of different p53 polymorphisms within the human population. Some of these polymorphisms are differentially distributed geographically—suggesting varied utility in different populations or climates (Beckman et al., 1994). One of these, the R72 polymorphism, can enhance fat accumulation and promote survival under nutrient starvation (Kung et al., 2016, 2017). This polymorphism is enriched in northern latitude populations, tracking nicely with selection based on altered necessities of survival in colder and more food-limited climes. However, whether such active positive selection caused this geographical distribution of the polymorphism is a matter of debate in the literature (Sucheston et al., 2011). Cells expressing a different p53 polymorphism (S47) show reduced ability to activate ferroptosis (Leu et al., 2019) and increased glycolysis (Barnoud et al., 2019b), correlating with reduced tumour suppressor activity of this variant (Jennis et al., 2016). Intriguingly, p53 S47 is found more frequently in populations of African descent, although why such polymorphisms would persist in these populations remains to be discovered (please see the review by Barnoud et al. (2019a) in this issue for more information on p53 S47 and other p53 variants). There is still a lot to learn, but already these observations highlight our growing appreciation of the importance of the metabolic functions of p53 beyond its usual cancer-centric conceptual framework.

*[We thank the members of the p53 and metabolism group for their helpful comments. Our work is funded by the Cancer Research UK grants C596/A17196 and C596/A26855 and supported by the Francis Crick Institute, which receives its core funding from Cancer Research UK (FC0010557), the UK Medical Research Council (FC0010557), and the Wellcome Trust (FC0010557).]*

## References

[ref1] Barnoud T., Parris J.L.D., and Murphy M.E. (2019a). Common genetic variants in the TP53 pathway and their impact on cancer. J. Mol. Cell Biol. 11, doi:10.1093/jmcb/mjz052 PMC6736421 31152665

[ref2] Barnoud T., Parris J.L.D., and Murphy M.E. (2019b). Tumor cells containing the African-centric S47 variant of TP53 show increased Warburg metabolism. Oncotarget 10, 1217–1223. 30838093 10.18632/oncotarget.26660 PMC6383823

[ref3] Beckman G., Birgander R., Sjalander A., et al. (1994). Is p53 polymorphism maintained by natural selection? Hum. Hered. 44, 266–270. 7927355 10.1159/000154228

[ref4] Bensaad K., Tsuruta A., Selak M.A., et al. (2006). TIGAR, a p53-inducible regulator of glycolysis and apoptosis. Cell 126, 107–120. 16839880 10.1016/j.cell.2006.05.036

[ref5] Chen X., Ko L.J., Jayaraman L., et al. (1996). p53 levels, functional domains, and DNA damage determine the extent of the apoptotic response of tumor cells. Genes Dev. 10, 2438–2451. 8843196 10.1101/gad.10.19.2438

[ref6] Derdak Z., Villegas K.A., Harb R., et al. (2013). Inhibition of p53 attenuates steatosis and liver injury in a mouse model of non-alcoholic fatty liver disease. J. Hepatol. 58, 785–791. 23211317 10.1016/j.jhep.2012.11.042 PMC3612370

[ref7] Duan L., Perez R.E., Chen L., et al. (2018). p53 promotes AKT and SP1-dependent metabolism through the pentose phosphate pathway that inhibits apoptosis in response to Nutlin-3a. J. Mol. Cell Biol. 10, 331–340. 29190376 10.1093/jmcb/mjx051 PMC6161407

[ref8] Eriksson M., Ambroise G., Ouchida A.T., et al. (2017). Effect of mutant p53 proteins on glycolysis and mitochondrial metabolism. Mol. Cell. Biol. 37, pii: e00328-17. 10.1128/MCB.00328-17 PMC5705820 28993478

[ref9] Feng Z., Hu W., de Stanchina E., et al. (2007). The regulation of AMPK β1, TSC2, and PTEN expression by p53: stress, cell and tissue specificity, and the role of these gene products in modulating the IGF-1–AKT–mTOR pathways. Cancer Res. 67, 3043–3053. 17409411 10.1158/0008-5472.CAN-06-4149

[ref10] Freed-Pastor W.A., Mizuno H., Zhao X., et al. (2012). Mutant p53 disrupts mammary tissue architecture via the mevalonate pathway. Cell 148, 244–258. 22265415 10.1016/j.cell.2011.12.017 PMC3511889

[ref11] Freed-Pastor W.A., and Prives C. (2012). Mutant p53: one name, many proteins. Genes Dev. 26, 1268–1286. 22713868 10.1101/gad.190678.112 PMC3387655

[ref12] Gnanapradeepan K., Basu S., Barnoud T., et al. (2018). The p53 tumor suppressor in the control of metabolism and ferroptosis. Front. Endocrinol. 9, 124. 10.3389/fendo.2018.00124 PMC5904197 29695998

[ref13] Gorrini C., Harris I.S., and Mak T.W. (2013). Modulation of oxidative stress as an anticancer strategy. Nat. Rev. Drug Discov. 12, 931–947. 24287781 10.1038/nrd4002

[ref14] Gudkov A.V., and Komarova E.A. (2010). Pathologies associated with the p53 response. Cold Spring Harb. Perspect. Biol. 2, a001180. 20595398 10.1101/cshperspect.a001180 PMC2890204

[ref15] Guo J.Y., and White E. (2016). Autophagy, metabolism, and cancer. Cold Spring Harb. Symp. Quant. Biol. 81, 73–78. 28209717 10.1101/sqb.2016.81.030981 PMC5521269

[ref16] Hardie D.G. (2014). AMPK—sensing energy while talking to other signaling pathways. Cell Metab. 20, 939–952. 25448702 10.1016/j.cmet.2014.09.013 PMC5693325

[ref17] Hu A., Zhang C., Wu R., et al. (2010). Glutaminase 2, a novel p53 target gene regulating energy metabolism and antioxidant function. Proc. Natl Acad. Sci. USA 107, 7455–7460. 20378837 10.1073/pnas.1001006107 PMC2867677

[ref18] Hu W., Feng Z., Teresky A.K., et al. (2007). p53 regulates maternal reproduction through LIF. Nature 450, 721–724. 18046411 10.1038/nature05993

[ref19] Humpton T.J., Hock A.K., Maddocks O.D.K., et al. (2018). p53-mediated adaptation to serine starvation is retained by a common tumour-derived mutant. Cancer Metab. 6, 18. 30524726 10.1186/s40170-018-0191-6 PMC6276204

[ref20] Humpton T.J., and Vousden K.H. (2016). Regulation of cellular metabolism and hypoxia by p53. Cold Spring Harb. Perspect. Med. 6, pii: a026146. 10.1101/cshperspect.a026146 PMC4930918 27371670

[ref21] Itahana Y., and Itahana K. (2018). Emerging roles of p53 family members in glucose metabolism. Int. J. Mol. Sci. 19, pii: E776. 10.3390/ijms19030776 PMC5877637 29518025

[ref22] Jennis M., Kung C.P., Basu S., et al. (2016). An African-specific polymorphism in the TP53 gene impairs p53 tumor suppressor function in a mouse model. Genes Dev. 30, 918–930. 27034505 10.1101/gad.275891.115 PMC4840298

[ref23] Jiang D., LaGory E.L., Kenzelmann Broz D., et al. (2015a). Analysis of p53 transactivation domain mutants reveals Acad11 as a metabolic target important for p53 pro-survival function. Cell Rep. 10, 1096–1109. 25704813 10.1016/j.celrep.2015.01.043 PMC4365998

[ref24] Jiang L., Kon N., Li T., et al. (2015b). Ferroptosis as a p53-mediated activity during tumour suppression. Nature 520, 57–62. 25799988 10.1038/nature14344 PMC4455927

[ref25] Jiang P., Du W., Mancuso A., et al. (2013). Reciprocal regulation of p53 and malic enzymes modulates metabolism and senescence. Nature 493, 689–693. 23334421 10.1038/nature11776 PMC3561500

[ref26] Jiang P., Du W., Wang X., et al. (2011). p53 regulates biosynthesis through direct inactivation of glucose-6-phosphate dehydrogenase. Nat. Cell Biol. 13, 310–316. 21336310 10.1038/ncb2172 PMC3110666

[ref27] Jones R.G., Plas D.R., Kubek S., et al. (2005). AMP-activated protein kinase induces a p53-dependent metabolic checkpoint. Mol. Cell 18, 283–293. 15866171 10.1016/j.molcel.2005.03.027

[ref28] Kamphorst J.J., Nofal M., Commisso C., et al. (2015). Human pancreatic cancer tumors are nutrient poor and tumor cells actively scavenge extracellular protein. Cancer Res. 75, 544–553. 25644265 10.1158/0008-5472.CAN-14-2211 PMC4316379

[ref29] Khan M.R., Xiang S., Song Z., et al. (2017). The p53-inducible long noncoding RNA TRINGS protects cancer cells from necrosis under glucose starvation. EMBO J. 36, 3483–3500. 29046333 10.15252/embj.201696239 PMC5709729

[ref30] Kung C.P., Leu J.I., Basu S., et al. (2016). The P72R polymorphism of p53 predisposes to obesity and metabolic dysfunction. Cell Rep. 14, 2413–2425. 26947067 10.1016/j.celrep.2016.02.037 PMC4926645

[ref31] Kung C.P., Liu Q., and Murphy M.E. (2017). The codon 72 polymorphism of p53 influences cell fate following nutrient deprivation. Cancer Biol. Ther. 18, 484–491. 28475405 10.1080/15384047.2017.1323595 PMC5639853

[ref32] Labuschagne C.F., Zani F., and Vousden K.H. (2018). Control of metabolism by p53—cancer and beyond. Biochim. Biophys. Acta Rev. Cancer 1870, 32–42. 29883595 10.1016/j.bbcan.2018.06.001 PMC6102416

[ref33] Leu J.I., Murphy M.E., and George D.L. (2019). Mechanistic basis for impaired ferroptosis in cells expressing the African-centric S47 variant of p53. Proc. Natl Acad. Sci. USA 116, 8390–8396. 30962386 10.1073/pnas.1821277116 PMC6486733

[ref34] Li L., Mao Y., Zhao L., et al. (2019). p53 regulation of ammonia metabolism through urea cycle controls polyamine biosynthesis. Nature 567, 253–256. 30842655 10.1038/s41586-019-0996-7

[ref35] Liu D., and Xu Y. (2011). p53, oxidative stress, and aging. Antioxid. Redox Signal. 15, 1669–1678. 21050134 10.1089/ars.2010.3644 PMC3151427

[ref36] Liu Y., He Y., Jin A., et al. (2014). Ribosomal protein—Mdm2—p53 pathway coordinates nutrient stress with lipid metabolism by regulating MCD and promoting fatty acid oxidation. Proc. Natl Acad. Sci. USA 111, E2414–E2422. 24872453 10.1073/pnas.1315605111 PMC4060669

[ref37] Long J.S., Crighton D., O'Prey J., et al. (2013). Extracellular adenosine sensing—a metabolic cell death priming mechanism downstream of p53. Mol. Cell 50, 394–406. 23603120 10.1016/j.molcel.2013.03.016

[ref38] Lowman X.H., Hanse E.A., Yang Y., et al. (2019). p53 promotes cancer cell adaptation to glutamine deprivation by upregulating Slc7a3 to increase arginine uptake. Cell Rep. 26, 3051–3060.e4. 30865893 10.1016/j.celrep.2019.02.037 PMC6510239

[ref39] Mayers J.R., and Vander Heiden M.G. (2017). Nature and nurture: what determines tumor metabolic phenotypes? Cancer Res. 77, 3131–3134. 28584183 10.1158/0008-5472.CAN-17-0165

[ref40] Moon S.H., Huang C.H., Houlihan S.L., et al. (2019). p53 represses the mevalonate pathway to mediate tumor suppression. Cell 176, 564–580.e519. 30580964 10.1016/j.cell.2018.11.011 PMC6483089

[ref41] Ou Y., Wang S.J., Jiang L., et al. (2015). p53 protein-mediated regulation of phosphoglycerate dehydrogenase (PHGDH) is crucial for the apoptotic response upon serine starvation. J. Biol. Chem. 290, 457–466. 25404730 10.1074/jbc.M114.616359 PMC4281747

[ref42] Park J.Y., Wang P.Y., Matsumoto T., et al. (2009). p53 improves aerobic exercise capacity and augments skeletal muscle mitochondrial DNA content. Circ. Res. 105, 705–712. 19696408 10.1161/CIRCRESAHA.109.205310 PMC2761626

[ref43] Pavlova N.N., and Thompson C.B. (2016). The emerging hallmarks of cancer metabolism. Cell Metab. 23, 27–47. 26771115 10.1016/j.cmet.2015.12.006 PMC4715268

[ref44] Porteiro B., Diaz-Ruiz A., Martinez G., et al. (2013). Ghrelin requires p53 to stimulate lipid storage in fat and liver. Endocrinology 154, 3671–3679. 23832961 10.1210/en.2013-1176

[ref45] Prokesch A., Graef F.A., Madl T., et al. (2017). Liver p53 is stabilized upon starvation and required for amino acid catabolism and gluconeogenesis. FASEB J. 31, 732–742. 27811061 10.1096/fj.201600845R PMC5240663

[ref46] Rabinowitz J.D., and White E. (2010). Autophagy and metabolism. Science 330, 1344–1348. 21127245 10.1126/science.1193497 PMC3010857

[ref47] Rosenfeldt M.T., O'Prey J., Morton J.P., et al. (2013). p53 status determines the role of autophagy in pancreatic tumour development. Nature 504, 296–300. 24305049 10.1038/nature12865

[ref48] Sucheston L., Witonsky D.B., Hastings D., et al. (2011). Natural selection and functional genetic variation in the p53 pathway. Hum. Mol. Genet. 20, 1502–1508. 21266458 10.1093/hmg/ddr028 PMC3063984

[ref49] Suzuki S., Tanaka T., Poyurovsky M.V., et al. (2010). Phosphate-activated glutaminase (GLS2), a p53-inducible regulator of glutamine metabolism and reactive oxygen species. Proc. Natl Acad. Sci. USA 107, 7461–7466. 20351271 10.1073/pnas.1002459107 PMC2867754

[ref50] Tajan M., Hock A.K., Blagih J., et al. (2018). A role for p53 in the adaptation to glutamine starvation through the expression of SLC1A3. Cell Metab. 28, 721–736. 30122553 10.1016/j.cmet.2018.07.005 PMC6224545

[ref51] Tarangelo A., Magtanong L., Bieging-Rolett K.T., et al. (2018). p53 suppresses metabolic stress-induced ferroptosis in cancer cells. Cell Rep. 22, 569–575. 29346757 10.1016/j.celrep.2017.12.077 PMC5791910

[ref52] Tran T.Q., Lowman X.H., Reid M.A., et al. (2017). Tumor-associated mutant p53 promotes cancer cell survival upon glutamine deprivation through p21 induction. Oncogene 36, 1991–2001. 27721412 10.1038/onc.2016.360 PMC5383530

[ref53] Warfel N.A., and El-Deiry W.S. (2013). p21WAF1 and tumourigenesis: 20 years after. Curr. Opin. Oncol. 25, 52–58. 23159848 10.1097/CCO.0b013e32835b639e

[ref54] Wu D., and Prives C. (2018). Relevance of the p53—MDM2 axis to aging. Cell Death Differ. 25, 169–179. 29192902 10.1038/cdd.2017.187 PMC5729541

[ref55] Xie Y., Zhu S., Song X., et al. (2017). The tumor suppressor p53 limits ferroptosis by blocking DPP4 activity. Cell Rep. 20, 1692–1704. 28813679 10.1016/j.celrep.2017.07.055

[ref56] Xu J., Wang J., Hu Y., et al. (2014). Unequal prognostic potentials of p53 gain-of-function mutations in human cancers associate with drug-metabolizing activity. Cell Death Dis. 5, e1108. 10.1038/cddis.2014.75 PMC3973211 24603336

[ref57] Zawacka-Pankau J., Grinkevich V.V., Hunten S., et al. (2011). Inhibition of glycolytic enzymes mediated by pharmacologically activated p53: targeting Warburg effect to fight cancer. J. Biol. Chem. 286, 41600–41615. 21862591 10.1074/jbc.M111.240812 PMC3308870

[ref58] Zhang C., Liu J., Liang Y., et al. (2013). Tumour-associated mutant p53 drives the Warburg effect. Nat. Commun. 4, 2935. 24343302 10.1038/ncomms3935 PMC3969270

